# Detection of Gastric Cancer with Fourier Transform Infrared Spectroscopy and Support Vector Machine Classification

**DOI:** 10.1155/2013/942427

**Published:** 2013-08-13

**Authors:** Qingbo Li, Wei Wang, Xiaofeng Ling, Jin Guang Wu

**Affiliations:** ^1^School of Instrumentation Science and Opto-Electronics Engineering, Precision Opto-Mechatronics Technology Key Laboratory of Education Ministry, Beihang University, Xueyuan Road Number 37, Haidian District, Beijing 100191, China; ^2^Department of General Surgery, Third Hospital, Peking University, Beijing 100083, China; ^3^College of Chemistry and Molecular Engineering, Peking University, Beijing 100871, China

## Abstract

Early diagnosis and early medical treatments are the keys to save the patients' lives and improve the living quality. Fourier transform infrared (FT-IR) spectroscopy can distinguish malignant from normal tissues at the molecular level. In this paper, programs were made with pattern recognition method to classify unknown samples. Spectral data were pretreated by using smoothing and standard normal variate (SNV) methods. Leave-one-out cross validation was used to evaluate the discrimination result of support vector machine (SVM) method. A total of 54 gastric tissue samples were employed in this study, including 24 cases of normal tissue samples and 30 cases of cancerous tissue samples. The discrimination results of SVM method showed the sensitivity with 100%, specificity with 83.3%, and total discrimination accuracy with 92.2%.

## 1. Introduction

Cancer is a disease that does great harms to the health of human beings. The development of tumor is a multistep, complex process, which is affected by many factors. By far, there have been few effective ways to cure the cancer. Thus, the survival of patients depends largely on the detection of cancer at an early stage. It is of great importance to explore the early cancer diagnosis method. But before the changes in cell morphology can be seen under light microscope, millions of cancer cells have already existed. In the process of carcinogenesis, nuclear acids, proteins, carbohydrates, and other biomolecules generate significant changes in their molecular structures. Fourier transform infrared (FT-IR) spectroscopy, as an effective tool for investigating chemical changes at molecular level, has been utilized to detect carcinoma. This method could detect human tissues directly without sample pretreatment, so the operation is simple, time saving, and convenient compared with the gene expression-based method. At present, with the development of biospectroscopy and spectral analysis technology, the application of FT-IR spectroscopy in distinguishing malignant tissues from normal ones has become a focus [1–5]. Also, great progresses have been made in the research of cancer detection using FT-IR spectroscopy. Some institutes, such as College of Chemistry and Molecular Engineering in Peking University in China, have got several achievements to a certain extent [6–11]. 

In this paper, the spectra of gastric tissues were collected by FT-IR spectrometer. In order to eliminate the high-frequency random noise, baseline drift, and light scattering, spectra preprocessing methods of smoothing and standard normal variate (SNV) were used. After spectra preprocessing, the spectra are presented with a high signal-to-noise ratio. In order to achieve a high discrimination accuracy of gastric cancer diagnosis, SVM method was adopted to classify the spectra of normal gastric tissues and cancerous gastric tissues.

## 2. Materials and Methods

### 2.1. Tissue Specimens

A total of 54 gastric tissues were obtained from the Surgery Department of the Third Hospital of Peking University, China. The samples were washed by distilled water and divided into two equal parts: one part for pathological study, the other part for FT-IR spectroscopic measurement. According to the results from the pathology diagnosis, the studied samples consisted of 30 cases of cancer tissues and 24 cases of normal tissues.

### 2.2. Acquisition of FT-IR Spectra of Gastric Tissues

The FT-IR spectra of samples were obtained on a Nicolet Magna 750 II FT-IR spectrometer. Spectra-Tech mid-IR optical fiber was utilized. Scan range is from 4000 cm^−1^ to 900 cm^−1^ with a resolution of 4 cm^−1^. 

### 2.3. Spectra Preprocessing Method

First, smoothing is utilized to filter high-frequency noise in FT-IR spectra. Then, the absorption band of CO_2_ is substituted with a straight line, which contains little useful information for measurement. Last, standard normal variate (SNV) method [12, 13] is adopted to weaken the effect to the accuracy of modeling from the differences of sample shape, size, density, and nonspecific scatter at the surface of the samples. 

Each spectrum was preprocessed by SNV; the spectroscopic data of sample *i* at wavenumber *k* could be standard normalized as (1):

(1) $$\begin{array}{c} x_{ik,\text{SNV}}=\frac{x_{i,k}-{\overset{-}{x}}_{i}}{\sqrt{\sum_{k=1}^{p}{\left(x_{i,k}-{\overset{-}{x}}_{i}\right)}^{2}}}{\left(p-1\right)}^{1/2}, \end{array}$$

where ${\overset{-}{x}}_{i}$ represents the average of spectroscopic data of sample *i*, while *p* is the number of wavelength, and (*p* − 1) is the freedom degrees. 

### 2.4. Discrimination Analysis Method

Support vector machine (SVM) is a kernel-based learning method rooted in structural risk minimization [14], and it is based on the concept of decision planes that define decision boundaries. A decision plane is one that separates between a set of objects having different class memberships. SVM has shown to be capable of handling high-dimensional data well, as its performance bounds on classification error do not explicitly depend on the dimensionality of the input data [15]. The principle is explained as follows [16].

For labeled training data of the form (*x*_*i*_, *y*_*i*_) *i* ∈ {1,…, *l*}, where *x*_*i*_ is an *n*-dimensional feature vector and *y* ∈ {−1,1} the labels, a decision function is found representing a separating hyperplane defined as (2):

(2) $$\begin{array}{c} f\left(x\right)=\left(\langle w,\phi \left(x_{i}\right)\rangle +b\right), \end{array}$$

where *w* is the weight vector, *b* is the bias value, and *ϕ*(*x*) is the kernel function. By projecting the data using a mapping *ϕ*(*x*), nonlinear decision boundaries in the input data space can be obtained. The separating hyperplane is found by maximizing distances to its closest data points, embedding it in a large margin which is defined by support vectors. Finding the hyperplane while maximizing the margin is formulated as the following optimization problem:

(3) min⁡⁡12wTw+C∑i=1Nξisubject  to:  yi(〈w,ϕ(xi)〉+b)≥1−ξi,           ξi≥0, (i=1,…,N),

where *C* is the cost parameter constant, *ξ*_*i*_ is parameter for handling nonseparable data, and the index *i* labels the *N* training cases. Note that *y* ∈ {−1,1} is the class labels and *x*_*i*_ is the independent variables. 

The optimization problem can be efficiently solved using an equivalent formulation to (3) using Lagrangian multipliers. In this formulation, data are represented exclusively by dot products, which can be replaced with a kernel function *K*(*x*, *x*′) = *ϕ*(*x*)^*T*^ *ϕ*(*x*′), allowing large margin separation in the kernel space. The choice of the kernel function determines the mapping *ϕ*(*x*) of the input data into a higher dimensional feature space, in which the linear separating hyperplane is found (see also (2)). In this paper, nonlinear classification using the Gaussian kernel as (4) was investigated:

(4) $$\begin{array}{c} K\left(x,x_{i}\right)=\exp\left(-\frac{{||x-x_{i}||}^{2}}{\sigma }\right). \end{array}$$

It comprises one free parameter, the kernel width *σ*, which controls the amount of local influence of support vectors on the decision boundary. 

## 3. Results and Discussion

### 3.1. FT-IR Spectra of Gastric Tissues

**Figure 1:**
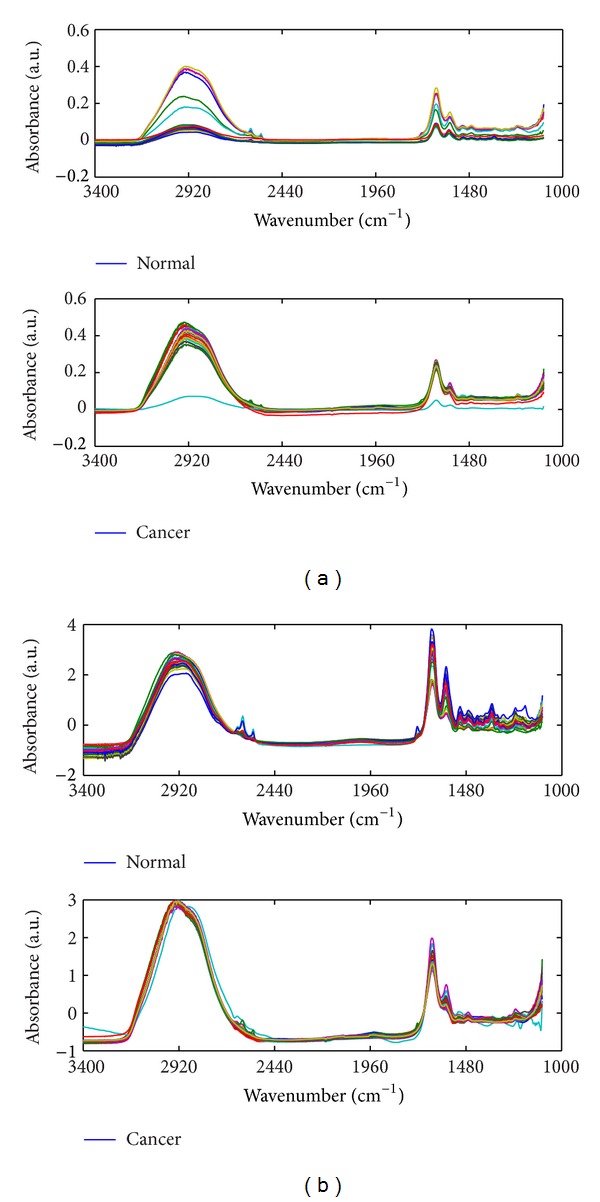
The FTIR spectra of normal and malignant gastric tissues. (a) Original spectra; (b) preprocessed spectra with smoothing and SNV.

The original FT-IR spectra (Figure 1(a)) were got; from it, it would be seen that the spectra obtained from two different experiments divided into two clusters. Contrast to Figure 1(b) which showed the spectra preprocessed with SVN, it would be found that low-frequency noise and baseline drift had been corrected with preprocessing. After spectra preprocessing, the differences between FT-IR spectra of normal gastric tissues and cancerous gastric tissues are more pronounced, and the boundary of two clusters is also clearer. The relevant information of FT-IR spectra has been explained in the previous paper [7, 10].

### 3.2. Discrimination Analysis

Before discrimination, resampling half mean (RHM) [17] method is adopted to eliminate the outliers, and the time of resampling process is set 500. The RHM scores of 54 gastric tissue samples are shown in Table 1, it could be seen that the samples Number 11, 18, and 19 were outliers, which would be removed.

After removing the outliers, discrimination model was built for classification of cancer and normal tissue samples, and leave-one-out cross validation (LOOCV) was utilized to evaluate the discrimination results of SVM method. The LOOCV method attempts to predict the data of the unknown sample with the data of training sample set. One sample was randomly selected and excluded from the training set. The selected sample regarded is as an unknown one, and then the sample is classified using the model built with the rest training samples. Record the result. Repeat the above course until all samples had been selected for once and only for once.

The discrimination results were shown in Table 2, which displays that among the 24 cases of normal tissue samples, 20 cases were correctly distinguished, while 4 samples were mis-judged; among the 27 cases of cancer tissue samples, all cases were well judged. The total accuracy was 92.2%.

From Table 2, it could be determined that the sensitivity of cancer diagnosis was 100%, specificity of cancer diagnosis was 83.3%, prediction value of a positive test was 87.1%, and prediction value of a negative test was 100%.

## 4. Conclusions

In this paper, the FT-IR spectra of normal gastric tissues and cancerous gastric tissues are acquired. After spectra preprocessing of smoothing and SNV, the spectra with high signal-to-noise ratio are presented and achieve a high discrimination accuracy with 92.2% by using SVM method. This indicates that FT-IR spectroscopy with chemometrics method is reliable, practical, and could be easily implemented in gastric cancer diagnosis.

## Figures and Tables

**Table 1:** RHM scores of gastric tissue samples.

Number	Score	Number	Score	Number	Score	Number	Score
1	0	15	0	29	9	43	0
2	0	16	0	30	0	44	0
3	0	17	0	31	0	45	0
4	24	18	471	32	0	46	0
5	0	19	325	33	0	47	0
6	15	20	0	34	0	48	0
7	0	21	0	35	0	49	0
8	0	22	0	36	0	50	0
9	0	23	0	37	0	51	0
10	0	24	42	38	0	52	0
11	289	25	0	39	0	53	0
12	0	26	102	40	0	54	0
13	0	27	0	41	0		
14	1	28	0	42	0		

**Table 2:** Discrimination results using SVM method.

Pathologic analyzing result	SVM method result	
	Positive (T+)	Negative (T−)
Cancer (D+)	27	0
Normal (D−)	4	20
